# Dysmagnesemia with acute kidney injury among older adults: clinical characteristics and prognostic importance

**DOI:** 10.1007/s40520-024-02872-x

**Published:** 2024-11-14

**Authors:** Qinglin Li, Xin Hu, Guanggang Li, Dawei Li, Qiangguo Ao, Feihu Zhou

**Affiliations:** 1 Department of Critical Care Medicine, The First Medical Center, Chinese PLA General Hospital, Beijing, 100853 China; 2 Department of Critical Care Medicine, The Seventh Medical Center, Chinese PLA General Hospital, Beijing, 100700 China; 3 Department of Critical Care Medicine, The Sixth Medical Center, Chinese PLA General Hospital, Beijing, 100048 China; 4 Department of Geriatric Nephrology, The Second Medical Centre, National Clinical Research Centre for Geriatric Diseases, Chinese PLA General Hospital, Beijing, 100053 China

**Keywords:** Serum magnesium, Dysmagnesemia, Acute kidney injury, Older patients, Mortality

## Abstract

**Purpose:**

The relationship between dysmagnesemia and all-cause mortality probability in individuals with acute kidney injury (AKI) have not been investigated. In this study, we evaluated the correlation of varying magnesium levels with mortality in older adults undergoing AKI.

**Patients and methods:**

Older adults receiving treatment at the Chinese PLA General Hospital between 2007 and 2018 were retrospectively recruited. All-cause mortality was evaluated at four preset magnesium concentrations: <0.8, 0.8–0.9, 0.9–1.0, and ≥ 1.0 mmol/L. Using multivariable-adjusted Cox assessment, the all-cause mortality risk was approximated by setting the reference magnesium concentration at 0.8–0.9 mmol/L.

**Results:**

Totally 744 participants were enrolled, whose median age was 88 years, with most of them being male (94.2%). Among them, 184 patients were assigned into the < 0.8 mmol/L group, 156 into the 0.8–0.9 mmol/L group, 206 into the 0.9–1.0 mmol/L group, and 198 into the ≥ 1.0 mmol/L group. After 28 days, the mortality rates in the four strata were 26.6, 17.9, 17.5, and 37.4%, respectively. The corresponding mortalities after 90 days were 42.4, 23.7, 26.7, and 45.5%, respectively. Compared with patients who had magnesium levels of 0.8–0.9 mmol/L, those with magnesium levels < 0.8 mmol/L (*P* = 0.048), and ≥ 1.0 mmol/L (*P* < 0.001) exhibited higher 28-day mortalities. Significant correlations also showed that patients with magnesium levels < 0.8 mmol/L (*P* = 0.017) and ≥ 1.0 mmol/L (*P* < 0.001) were significantly related to the increased 90-day mortality.

**Conclusion:**

Magnesium levels outside the interval of 0.8–1.0 mmol/L were related to the higher risks of 28- and 90-day mortalities among older adults with AKI.

## Introduction

As the life expectancy prolongs and demographic structure shifts in China, the elderly are expected to account for an increasingly higher proportion of the overall population. Older patients are highly likely to develop lethal electrolyte disorders owing to age, comorbidities, malnutrition, prolonged pharmaceutical use, and dietary inadequacies [1, 2]. As an essential mineral element in the human body, magnesium is the fourth richest cation after sodium, potassium, and calcium, and the second richest intracellular cation after potassium [3]. Several studies have indicated that hospital-acquired dysmagnesemia is common and significantly related to the longer duration of hospitalization and higher probability of death [4–6]. However, clinicians emphasize more on the sodium, potassium and calcium abnormalities, while tending to ignore magnesium electrolyte imbalance in routine clinical practice [7].

The kidney serves as a vital regulator for magnesium balance. Understandably, magnesium has been found to play a vital role in the pathophysiology of nephropathies. For example, previous reports have indicated that both admission hypermagnesemia and hypomagnesemia were associated with high risk of hospital-acquired AKI [8–10]. Hypomagnesemia is also linked to non-restoration of kidney function following an AKI episode [11, 12]. Meanwhile, the start of renal replacement therapy (RRT) for the most severe form of AKI may further condition magnesium serum levels. Therefore, assessing magnesium imbalance may provide additional prognostic information in terms of AKI patients.

Recently, a retrospective cohort study was performed to evaluate the correlation of sodium, potassium, and mortality in older patients with AKI. We demonstrated that sodium concentrations outside the interval of 130.0–141.9 mmol/L and/or potassium ≥ 4.10 mmol/L were related to short- and long-term mortalities [13, 14]. However, the synergistic or independent prognostic impact of abnormal magnesium concentrations is poorly investigated, and the normal range of magnesium concentrations appropriate for these patients is unclear. Therefore, when making decisions about older patients with AKI and dysmagnesemia, it is vital for clinicians to identify the clinically meaningful normal magnesium ranges.

## Patients and methods

A retrospective study was carried out in consecutive patients (≥ 75 years of age) who had healthy kidney function, and were hospitalized in the Geriatric Department of the Chinese PLA General Hospital (Beijing, China) from January 2007 to December 2018. The research protocol was approved by the Clinical Ethics Committee of the aforementioned hospital (No.: S2023–725-01). Since the present research was observational and retrospective, it was unnecessary to obtain written informed consent from each patient. In addition, all patient data were anonymized. This study was undertaken following the Declaration of Helsinki tenets. All admissions were screened and assessed for AKI and classified in line with the kidney disease: Improving Global Outcomes (KDIGO) guidelines.

Age, sex, and comorbidities which included coronary artery disease, chronic obstructive pulmonary disease (COPD), diabetes mellitus and hypertension were collected. The AKI etiology including hypovolemia, cardiovascular episodes, sepsis, nephrotoxic medication and surgery, and vital signs like diastolic and systolic blood pressures were reported. The received medical treatments were also recorded, including angiotensin-converting enzyme inhibitor/angiotensin receptor blocker, calcium antagonists, diuretics, beta-blockers, magnesium supplement, and mechanical ventilation. Other laboratory tests of interest included serum creatinine (Scr) levels at baseline and diagnosis of AKI, the levels of blood glucose, blood urea nitrogen (BUN), uric acid, serum electrolytes (sodium, potassium, calcium, phosphate, and magnesium), albumin, C-reactive protein, hemoglobin, and prealbumin.

Exclusion criteria were participants with a prior history of chronic kidney disease (CKD), those had a less than 48 h of hospitalization, those without Scr data or only single Scr test, those without adequate medical documents, and those who died within 48 h of hospitalization.

### Definitions

AKI identification and categorization were performed following the 2012 KDIGO-defined Scr criteria [15]. The estimated glomerular filtration rate (eGFR) at baseline was estimated using the CKD Epidemiology Collaboration formula [16]. Baseline Scr concentration was the most recent measurement taken 1–3 months before the hospitalization for AKI [17]. The Surviving Sepsis Campaign Bundle: 2018 update was used for the definition of sepsis [18]. 

For our study population, the median (25th–75th percentiles) magnesium level at the diagnosis of AKI was 0.9 mmol/L. The baseline clinical traits of our participant groups were stratified according to the magnesium concentration quartiles as follows: <0.8 mmol/L, 0.8–0.9 mmol/L, 0.9–1.0 mmol/L, and ≥ 1.0 mmol/L. Four magnesium intervals were evaluated, with 0.8–0.9 mmol/L set as the reference interval on the basis of analysis, demonstrating the lowest mortality risk in this range (Table 1).

**Table 1:** Demographic data stratification according to serum magnesium levels

Characteristic	AKI patients *n* = 744	< 0.8 mmol/L *n* = 184, 24.7	0.8–0.9 mmol/L *n* = 156, 21.0	0.9–1.0 mmol/L *n* = 206, 27.7	≥ 1.0 mmol/L *n* = 198, 26.6	*P*-value
Age (years)	88 (84–91)	89 (83–92)	87 (84–91)	87 (84–91)	87 (84–91)	0.475
Male sexr	701 (94.2)	166 (90.2)	150 (96.2)	191 (92.7)	194 (98.0)	0.006
Body mass index (kg/m^2^)	23.0 ± 3.1	22.6 ± 3.3	23.1 ± 3.1	23.0 ± 3.0	23.2 ± 3.1	0.115
Comorbidity						
Coronary disease	565 (75.9)	138 (75.0)	121 (77.6)	154 (74.8)	152 (76.8)	0.909
Hypertension	546 (73.4)	130 (70.7)	113 (72.4)	162 (78.6)	141 (71.2)	0.244
COPD	511 (68.7)	125 (67.9)	110 (70.5)	137 (66.5)	139 (70.2)	0.812
Diabetes	283 (38.0)	65 (35.3)	55 (35.3)	81 (39.3)	82 (41.4)	0.534
Baseline Scr (µmol/L)	72.0 (60.0–82.0)	66.0 (54.0–80.0)	74.0 (60.0–85.0)	75.0 (65.0–84.0)	72.0 (60.0–82.0)	< 0.001
Baseline eGFR (mL/min/1.73 m^2^)	78.7 (72.2–85.4)	81.8 (74.5–88.5)	78.3 (70.5–84.8)	77.0 (70.7–83.1)	78.7 (73.2–84.9)	< 0.001
Etiology of AKI						
Sepsis	312 (41.9)	86 (46.7)	55 (35.3)	80 (38.8)	91 (46.0)	0.081
Hypovolemia	159 (21.4)	36 (19.6)	33 (21.2)	45 (21.8)	45 (22.7)	0.895
Cardiovascular events	111 (14.9)	22 (12.0)	28 (17.9)	32 (15.5)	29 (14.6)	0.480
Nephrotoxicity	89 (12.0)	22 (12.0)	18 (11.5)	29 (14.1)	20 (10.1)	0.670
Surgery	49 (6.6)	12 (6.5)	12 (7.7)	15 (7.3)	10 (5.1)	0.745
Others	24 (3.2)	6 (3.3)	10 (6.4)	5 (2.4)	3 (1.5)	0.062
Clinical conditions						
Mean arterial pressure (mmHg)	78 ± 14	78 ± 14	80 ± 15	78 ± 13	77 ± 15	0.737
Oliguria	46 (6.2)	11 (6.0)	11 (7.1)	9 (4.4)	15 (7.6)	0.563
Mechanical ventilation	298 (40.1)	82 (44.6)	48 (30.8)	67 (32.5)	101 (51.0)	< 0.001
Pharmacotherapy						
ACEIs/ARBs	414 (55.6)	107 (58.2)	83 (53.2)	123 (59.7)	101 (51.0)	0.267
Beta-blockers	336 (45.2)	69 (37.5)	81 (51.9)	101 (49.0)	85 (42.9)	0.031
Calcium channel blockers	306 (41.1)	71 (38.6)	65 (41.7)	91 (44.2)	79 (39.9)	0.700
Diuretics	602 (80.9)	144 (78.3)	119 (76.3)	170 (82.5)	169 (85.4)	0.118
Magnesium supplement	446 (59.9)	113 (61.4)	76 (48.7)	123 (59.7)	134 (67.7)	0.004
Laboratory parameters						
Scr (µmol/L)	128.4. (115.0–145.0)	125.2 (111.2–143.1)	126.6 (113.2–141.9)	127.1 (116.0–141.1)	135.1 (121.0–153.1)	< 0.001
Peak Scr (µmol/L)	144.0 (124.0–206.4)	148.4 (121.3–208.9)	137.9 (117.3–160.9)	136.0 (124.0–179.1)	172.2 (132.7–264.2)	< 0.001
Blood urea nitrogen (mmol/L)	12.7 (8.9–20.7)	13.0 (8.9–22.0)	10.1 (7.9–16.2)	11.3 (8.7–17.7)	17.1 (11.4–26.0)	< 0.001
Uric acid (mmol/L)	366.5 (291.3–468.3)	354.8 (281.8–449.3)	355.0 (277.5–426.5)	371.1 (297.1–467.5)	407.6 (306.9–511.0)	0.001
Blood glucose (mmol/L)	7.4 (5.8–10.3)	7.6 (5.8–10.5)	7.0 (5.5–10.2)	7.3 (5.7–9.3)	7.9 (6.2–11.2)	0.028
Potassium (mmol/L)	4.2 (3.8–4.7)	4.1 (3.7–4.7)	4.1 (3.7–4.6)	4.1 (3.9–4.6)	4.2 (3.9–4.9)	0.052
Sodium (mmol/L)	140.0 (136.0–147.0)	139.0 (135.0–146.0)	139.0 (134.0–144.0)	140.0 (136.0–145.0)	144.0 (138.0–151.0)	< 0.001
Calcium (mmol/L)	2.2 (2.1–2.4)	2.1 (2.0–2.3)	2.2 (2.1–2.4)	2.2 (2.1–2.4)	2.2 (2.1–2.4)	0.001
Phosphate (mmol/L)	1.2 (0.9–1.4)	1.1 (0.8–1.4)	1.2 (1.0–1.4)	1.2 (1.0–1.4)	1.3 (1.0–1.6)	< 0.001
Magnesium (mmol/L)	0.9 (0.8–1.0)	0.7 (0.6–0.7)	0.9 (0.8–0.9)	1.0 (0.9–1.0)	1.1 (1.0–1.2)	< 0.001
C-reactive protein (mmol/L)	4.2 (1.9–9.6)	6.9 (2.3–13.7)	3.3 (1.5–8.4)	3.4 (1.7–7.3)	4.6 (2.1–9.6)	< 0.001
Albumin (g/L)	34.2 ± 5.6	31.8 ± 5.5	34.9 ± 4.7	35.3 ± 5.6	34.9 ± 5.7	< 0.001
Prealbumin (g/L)	176(134–229)	151(116–201)	189 (143–234)	196 (147–247)	176 (141–227)	< 0.001
Hemoglobin (g/L)	112 ± 22	106 ± 22	114 ± 21	113 ± 21	115 ± 24	< 0.001
AKI Stage						< 0.001
1	323 (43.4)	60 (32.6)	88 (56.4)	111 (53.9)	64 (32.3)	
2	190 (25.5)	56 (30.4)	36 (23.1)	44 (21.4)	54 (27.3)	
3	231 (31.0)	68 (37.0)	32 (20.5)	51 (24.8)	80 (40.49)	
Outcomes						
Renal replacement therap	4 (0.5)	2 (1.1)	1 (0.6)	0	1 (0.5)	0.383
28-day mortality	187 (25.1)	49 (26.6)	28 (17.9)	36 (17.5)	74 (37.4)	< 0.001
90-day mortality	260 (34.9)	78 (42.4)	37 (23.7)	55 (26.7)	90 (45.5)	< 0.001

*Note* Values are n (%), mean ± SD or median (inter-quartile range)

*Abbreviations* AKI, acute kidney injury; COPD, chronic obstructive pulmonary disease; eGFR, estimated glomerular fitration rate; Scr, serum creatinine; ACEIs/ARBs, angiotensin-converting enzyme inhibitor/angiotensin receptor blocker

### Outcomes

The all-cause mortalities within 28 and 90 days following the diagnosis of AKI were used as the outcomes of this study.

### Statistical analysis

Continuous variables were indicated as means ± standard deviations in case they were parametric, or as medians with interquartile ranges (25th–75th percentiles) if they were nonparametric. Categorical variables were expressed as percentages (%) or numbers (n). Group comparisons were performed by Kruskal–Wallis H test or ANOVA for continuous variables, while by Fisher’s exact or Pearson’s chi-square test for categorical variables. The Cox proportional hazards model was used to determine prognostic survival factors. Survival probability was approximated by the Kaplan–Meier estimator for the four magnesium intervals. Log-rank test was used for inter-group comparison of curves. Data were statistically assessed with SPSS 21.0 for Windows (SPSS Inc., IBM, Armonk, NY, USA). Differences were considered significant at *P* < 0.05.

## Results

### Study population

**Fig. 1:**
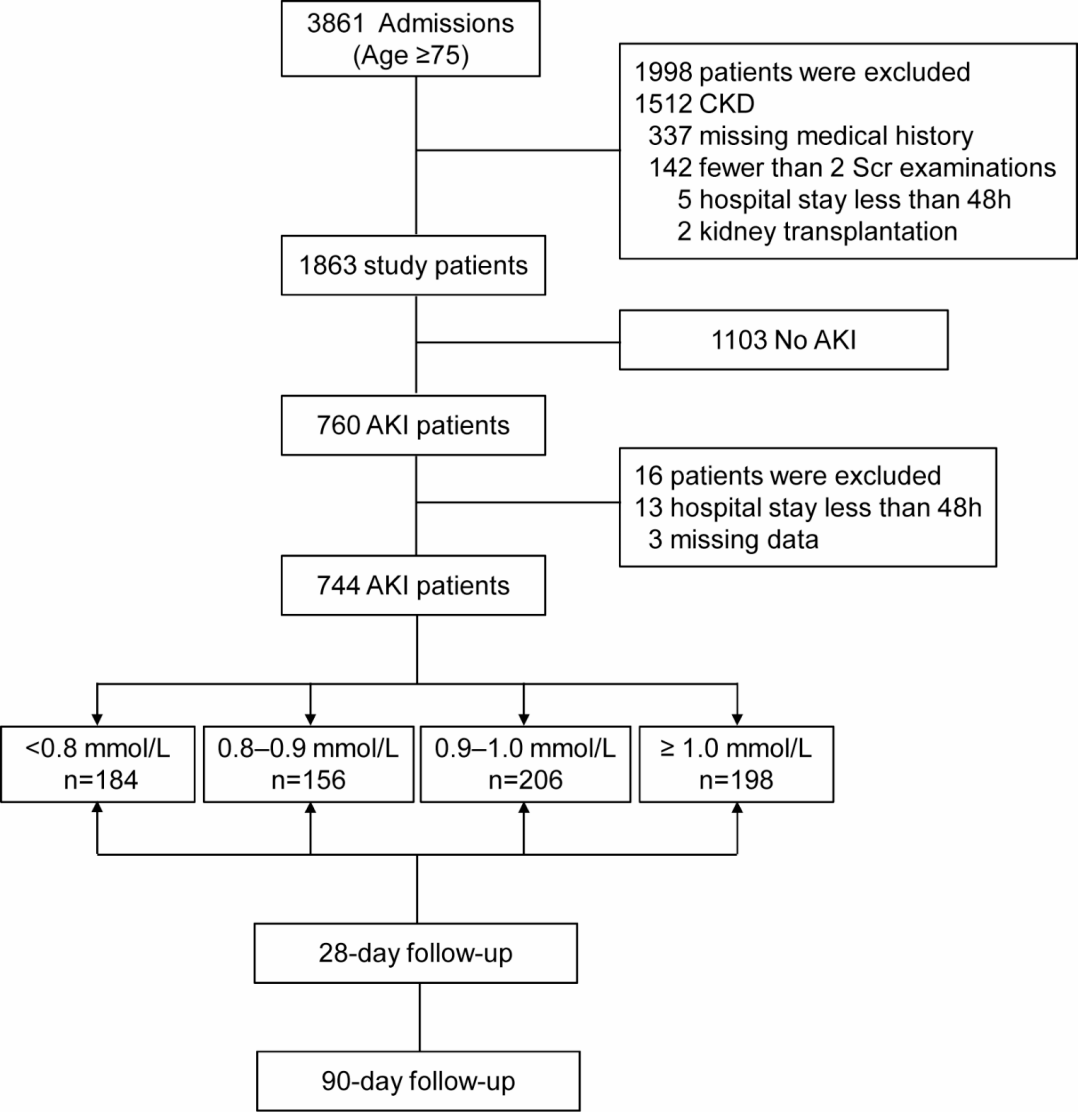
Flow chart of the inclusion and exclusion process of patients in this study

Among the 3861 older patients (≥ 75 years) screened during the research period, 760 had AKI, and 1998 were eliminated. In addition, 13 of the remaining 760 patients were eliminated due to hospitalization for less than 48 h. Three were eliminated due to insufficient data. Therefore, 744 patients with AKI were involved in the final analysis. The processes of patient selection and identification along with inclusion and exclusion criteria are presented in Fig. 1.

### Descriptive data

Table 1 provides more details of the baseline traits of the investigated population. These 744 participants had a median age of 88 years, and most of them were male (701/744, 94.2%). Among all the patients, 324 (43.4%) were diagnosed with stage 1 AKI, 190 (25.5%) with stage 2 AKI, and 231 (31.0%) with stage 3 AKI. In general, 187 (25.1%) patients died within 28 days, and 260 (34.9%) died within 90 days.

### General conditions and clinical characteristics according to magnesium levels

**Fig. 2:**
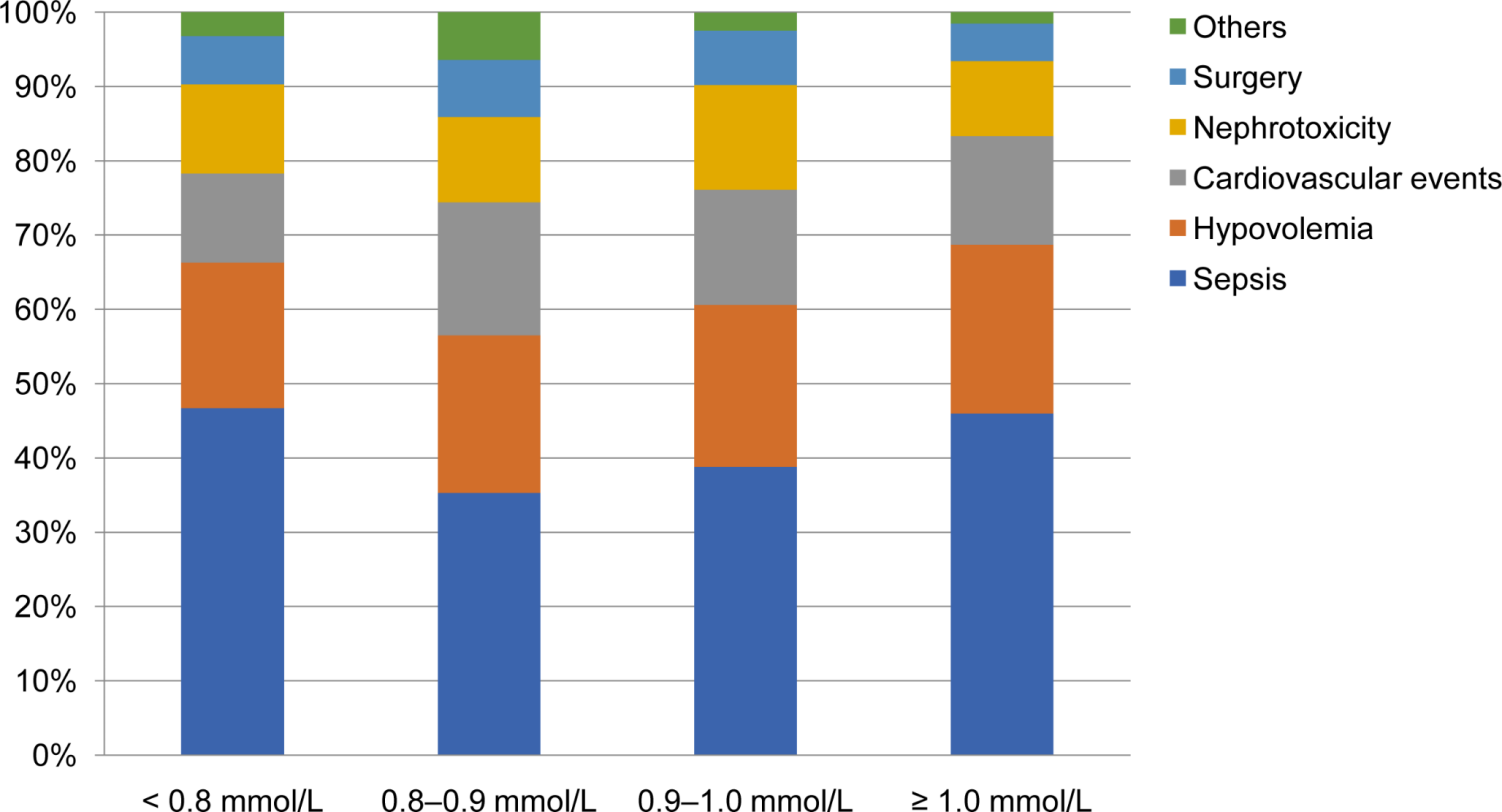
Percentage distribution of serum magnesium in AKI patients with different etiologies

The overall median magnesium concentration was 0.9 mmol/L (0.8–1.0 mmol/L) upon the diagnosis of AKI. Totally 184 participants were assigned into the < 0.8 mmol/L group, 156 into the 0.8–0.9 mmol/L group, 206 into the 0.9–1.0 mmol/L group, and 198 into the ≥ 1.0 mmol/L group. Figure 2 shows the percentage distribution of serum magnesium in AKI patients with different etiologies upon the diagnosis of AKI. Regardless of the serum magnesium concentration, the top five etiologies of AKI included sepsis, hypovolemia, cardiovascular events, nephrotoxicity, and surgery. Among sepsis-induced AKI patients, more commonly observed serum levels of magnesium were < 0.8 mmol/L and ≥ 1.0 mmol/L than 0.8–1.0 mmol/L.

Significant inter-group disparities were observed in male sex, baseline Scr, baseline eGFR, demand for mechanical ventilation, Scr, peak Scr, BUN, blood glucose, uric acid, calcium, sodium, phosphate, hemoglobin, albumin, prealbumin, C-reactive protein, and AKI stage upon the diagnosis of AKI. The significant correlations of serum magnesium concentration with both 28- and 90-day mortalities were also observed (*P* < 0.001). The 28-day mortalities in the four magnesium interval groups from the lowest to the highest were 26.6, 17.9, 17.5, and 37.4%, respectively, while the corresponding 90-day mortalities were 42.4, 23.7, 26.7, and 45.5%, respectively.

### Survival analysis

**Fig. 3:**
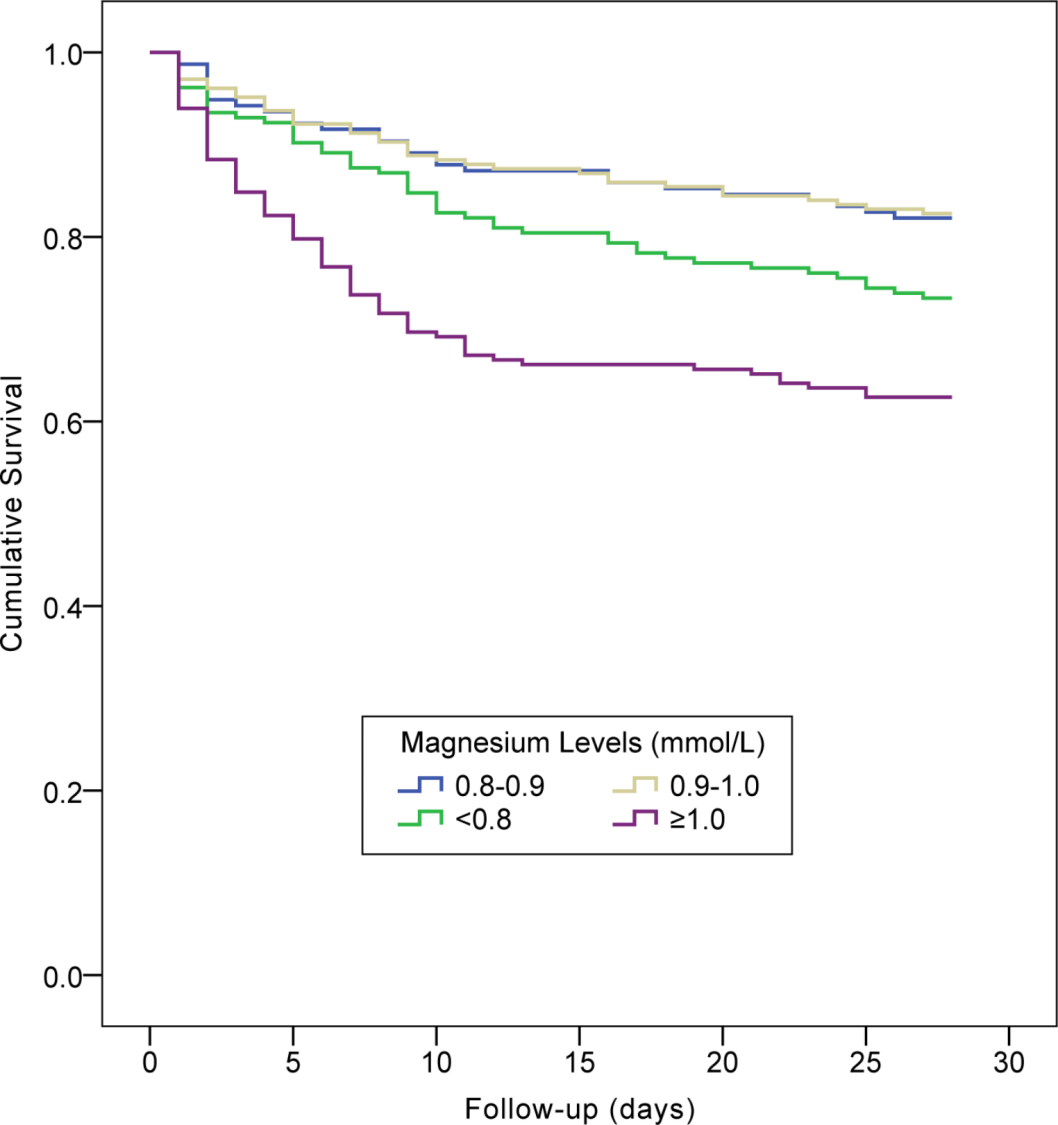
Kaplan–Meier plot of cumulative 28-day mortality rates across strata of serum magnesium concentration (log rank test: *P* < 0.001)

**Fig. 4:**
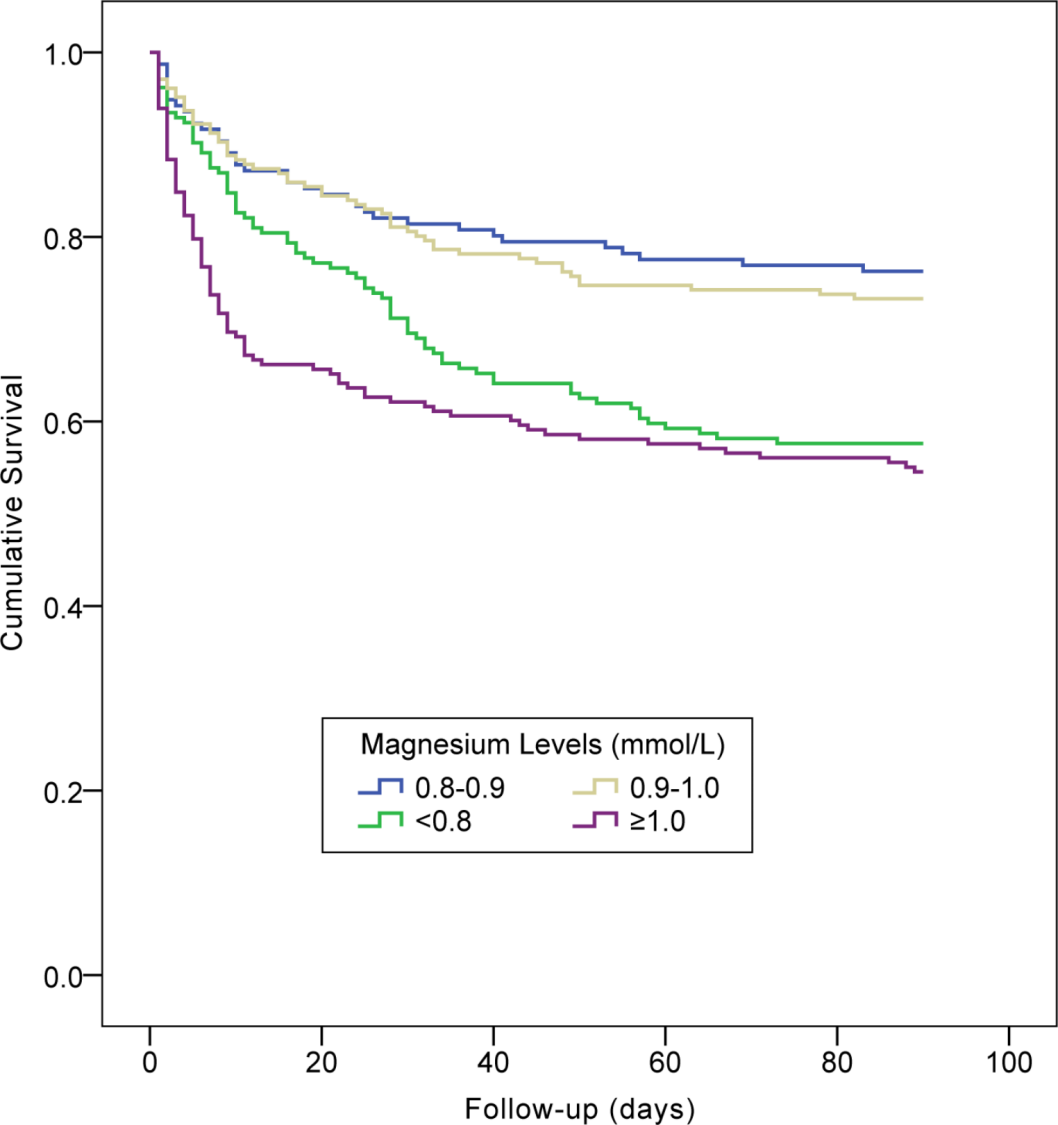
Kaplan–Meier plot of cumulative 90-day mortality rates across strata of serum magnesium concentration (log rank test: *P* < 0.001)

Over the 28-day follow-up period, 187 (25.1%) of the investigated population died. As shown in Table 2, among the all-cause deaths, 15.0%, 19.3%, 26.2%, and 39.6% patients had magnesium levels of 0.8–0.9, 0.9–1.0, < 0.8, and ≥ 1.0 mmol/L, respectively. Figure 3 displays the survival curves for 28-day all-cause mortalities across strata of serum magnesium. Over a 90-day follow-up period, 260 (34.9%) died. Among all the deaths from AKI, 14.2%, 21.2%, 30.0%, and 34.6% patients had magnesium levels of 0.8–0.9, 0.9–1.0, < 0.8, and ≥ 1.0 mmol/L, respectively (Table 2). Figure 4 presents the survival curves for 90-day all-cause mortalities across strata of serum magnesium.

**Table 2:** Association of categories of serum magnesium levels with 28-day and 90-day mortality

Characteristic	28-day outcomes				90-day outcomes		
	Nonsurvivors *n* = 187 (25.1)	Survivors *n* = 557 (74.9)	*P*-value		Nonsurvivors *n* = 260 (34.9)	Survivors *n* = 484 (65.1)	*P*-value
Magnesium levels (mmol/L)			< 0.001				< 0.001
0.8–0.9	28 (15.0)	128 (23.0)			37 (14.2)	119 (24.6)	
0.9–1.0	36 (19.3)	170 (30.5)			55 (21.2)	151 (31.2)	
< 0.8	49 (26.2)	135 (24.2)			78 (30.0)	106 (21.9)	
≥ 1.0	74 (39.6)	124 (22.3)			90 (34.6)	108 (22.3)	

*Note* Values are n (%)

During the multivariable-adjusted analysis, individuals with magnesium concentrations < 0.8 mmol/L (HR: 1.599; 95% CI: 1.004–2.546; *P* = 0.048), and ≥ 1.0 mmol/L (HR: 2.553; 95%CI: 1.651–3.947; *P* < 0.001) exhibited relatively higher 28-day mortalities. Significant association of magnesium concentration with 90-day mortality was also found: individuals having magnesium concentrations < 0.8 mmol/L (HR: 1.616; 95% CI: 1.090–2.395; *P* = 0.017), and ≥ 1.0 mmol/L (HR: 2.268; 95%CI: 1.534–3.354; *P* < 0.001) were significantly related to the increased 90-day mortality (Table 3).

**Table 3:** Multivariate Cox proportional hazard model analysis of risk factors for mortality

Risk factor		28-day mortality				90-day mortality	
	HR	95%CI	*P*-value		HR	95%CI	*P*-value
Body mass index	0.941	0.898–0.986	0.011		0.932	0.894–0.972	0.001
ACEI/ARBs	0.450	0.334–0.607	< 0.001		0.586	0.457–0.751	< 0.001
MAP	–	–	–		0.967	0.959–0.975	< 0.001
Hemoglobin	–	–	–		0.989	0.983–0.995	< 0.001
Mechanical ventilation	–	–	–		2.675	2.023–3.536	< 0.001
Magnesium levels (mmol/L)			< 0.001				< 0.001
0.8–0.9	Reference	Reference	Reference		Reference	Reference	Reference
0.9–1.0	1.037	0.632–1.699	0.887		1.200	0.790–1.824	0.393
< 0.8	1.599	1.004–2.546	0.048		1.616	1.090–2.395	0.017
≥ 1.0	2.553	1.651–3.947	< 0.001		2.268	1.534–3.354	< 0.001

*Abbreviations* ACEIs/ARBs, angiotensin-converting enzyme inhibitor/angiotensin receptor blocker; MAP, mean arterial pressure; CI, confdence interval; HR, hazard ratio

## Discussion

Despite being a vital type of cation in the human body, magnesium is somehow not comprehensively considered in clinical and preclinical analyses. Since the clinically used normal range of serum magnesium concentrations 0.7–1.0 mmol/L is primarily set according to healthy participants aged 18–74 years, whether this range is applicable to older adults with complex comorbidities remains unclear [19–21]. This study indicated a frequent incidence of dysmagnesemia among the very advanced-age hospitalized AKI patients, where 49% of the patients had an optimal magnesium level (0.8–1.0 mmol/L). After multivariable adjustment, magnesium concentrations of < 0.8 and ≥ 1.0 mmol/L were still the significant predictors for mortality.

Hypomagnesemia has been previously reported to be the prevailing electrolyte imbalance in clinical practice, which ranges from 9 to 65% and is particularly observed in the intensive care unit (ICU) patients [7]. Different from hypomagnesemia, hypermagnesemia is a less common condition, with rates ranging from 1.3–23.6%.^1,5,6,22^ However, the definition of hypermagnesemia varies from literature to literature, with lower limits set at 0.9, 1.0, 1.1, or 1.2 mmol/L. Based on our study, the mortalities of both hypomagnesemia and hypermagnesemia were around 25% and the rate of dysmagnesemia was approximately 50%. Among the older population, the mortality of electrolyte imbalance is shown to be higher due to organ degeneration, compromised physiologic reserves, infirmity, as well as higher prevalence of dysfunction and disability [1, 22]. The function of kidneys is to maintain water homeostasis, while AKI can amplify the impact of dysmagnesemia with clinical consequences. AKI disrupts the physiological modulation of electrolyte homeostasis by interfering with tubular functions, which may be more pronounced in older adults [19]. 

Magnesium is a vital cation maintaining normal cellular physiology. Changes in serum magnesium levels can cause dysfunction of multiple bodily organs and tissues, including the respiratory, circulatory systems and muscles. A few previous studies have explored the association of serum magnesium with AKI probability in other populations, such as critically ill children, cancer patients, acute pancreatitis, and those receiving cardiac surgery [8–10, 23]. The impact of serum magnesium ions on the prognosis of AKI patients has seldom been reported. Previous scholars have mainly explored the association of serum magnesium with the outcome of hospitalized patients. Due to the relatively rare occurrence of hypermagnesemia, relevant literature has mostly reported the association of hypomagnesemia with mortality, while different reports have yielded different results. In the studies of Al Alawi AM et al. [24], Cheungpasitporn W et al. [5], and Wolf et al. [25], the mortality of hypomagnesaemic patients was shown to be significantly higher than those having normal blood magnesium levels. However, Cheungpasitporn W et al. reported no mortality difference between normomagnesemic and hypomagnesemic hospitalized patients [4]. Similarly, Angkananard T et al. revealed no mortality disparity between normomagnesemic and hypomagnesemic heart failure patients [26]. In this study, the shortened survivals were found for subjects with both low (< 0.8 mmol/L) and high (≥ 1.0 mmol/L) magnesium levels. However, the highest magnesium range (≥ 1.0 mmol/L) was the strongest independent predictor for 28- and 90-day mortalities, with a 2-fold increase in both risks compared with that in older normomagnesemic individuals.

Magnesium has been referred to as “forgotten electrolyte”, since although magnesium alterations are frequently seen, it is challenging to diagnose dysmagnesemia considering its rarity and lack of regular magnesium level monitoring [20, 27]. In addition, the symptoms and signs are nonspecific, usually resulting in delayed diagnosis. The prognosis of dysmagnesemia depends on the degree of electrolyte imbalance. Favorable prognosis of older AKI sufferers is expected in those who have magnesium level within 0.8–1.0 mmol/L, while those with magnesium levels < 0.8 and ≥ 1.0 mmol/L are at an increased risk of death. Therefore, timely magnesium identification and management exert an essential role in preventing potentially lethal complications, particularly in older AKI patients.

Generally, RRT is provided as a supportive treatment to AKI patients. The current KDIGO guidelines recommend the initiation of RRT emergently if there are life-threatening complications of AKI. However, the timing of RRT and modality selected for the most severe form of AKI may further condition magnesium levels [28]. In older AKI patients with the need for continuous RRT, other causes of hypomagnesemia may be added, especially when regional citrate anticoagulation is used [29]. The chelation of cations by citrate results in hypomagnesemia in case of inappropriate substitution [30]. In this study, only 4 patients required RRT, and believe that the impact on magnesium level is relatively small. Decisions about RRT initiation in older AKI patients are influenced by several factors: AKI severity, blood vessel volume, electrolyte and acid–base status, urine output, hemodynamics, nutritional status, and attending physicians’ preferences. Our study showed that magnesium concentrations of < 0.8 and ≥ 1.0 mmol/L were associated with a significantly increased 28-day and 90-day risk of death. Clinicians may not perform strict electrolyte management in patients with magnesium levels ranging from 0.7 to 0.8 mmol/L, because these AKI patients have normal magnesium levels. Our results have indicated that mortality is increased even in patients with normal magnesium levels. This reminds clinicians to evaluate other conditions throughout the body of older patients with magnesium levels within the normal range, and then optimize the treatment such as the timing for RRT.

### Limitations

However, this study still has the following limitations. At first, our single-center investigation was observational and retrospective in nature, only comprising older-patient admissions. We could only establish a correlation instead of a causal relationship. When applying patient records from other centers, different results could be obtained. Therefore, subject selection bias can not be neglected. Second, only the serum magnesium concentrations at the diagnosis of AKI were assessed, which are liable to change over time (including electrolyte therapy initiation). In future studies, the magnesium concentrations need to be significantly measured for more objective assessment of the effect of magnesium. Third, despite our attempt to account for several potential confounders in the multivariable analyses, it appears to be reasonable that our results are probably influenced by other unascertained variables. Fourth, we do not have accurate data on patients undergoing RRT, such as the timing of initiating RRT and modality selected. Considering that RRT can affect the patient’s electrolyte levels, this is one of our limitations. However, only 4 patients of our study required RRT, and we believe that this impact is relatively small.

## Conclusion

Dysmagnesemia with AKI is common, affecting approximately one-half in older patients. Magnesium levels outside the interval of 0.8–1.0 mmol/L are related to increased 28- and 90-day mortality probabilities, suggesting that maintaining a narrow range (0.8–1.0 mmol/L) in older AKI patients may be an applicable strategy.

## Data Availability

No datasets were generated or analysed during the current study.
